# Harnessing Cellular Immunity for Vaccination against Respiratory Viruses

**DOI:** 10.3390/vaccines8040783

**Published:** 2020-12-21

**Authors:** Nicholas W. Lukacs, Carrie-Anne Malinczak

**Affiliations:** 1 Department of Pathology, University of Michigan, Ann Arbor, MI 48109, USA; nlukacs@med.umich.edu; 2 Mary H. Weiser Food Allergy Center, University of Michigan, Ann Arbor, MI 48109, USA

**Keywords:** vaccine, RSV, SARS-CoV-2, respiratory viruses, cellular immunity, nanoparticles, virus-like particles, RNA

## Abstract

Severe respiratory viral infections, such as influenza, metapneumovirus (HMPV), respiratory syncytial virus (RSV), rhinovirus (RV), and coronaviruses, including severe acute respiratory syndrome coronavirus-2 (SARS-CoV-2), cause significant mortality and morbidity worldwide. These viruses have been identified as important causative agents of acute respiratory disease in infants, the elderly, and immunocompromised individuals. Clinical signs of infection range from mild upper respiratory illness to more serious lower respiratory illness, including bronchiolitis and pneumonia. Additionally, these illnesses can have long-lasting impact on patient health well beyond resolution of the viral infection. Aside from influenza, there are currently no licensed vaccines against these viruses. However, several research groups have tested various vaccine candidates, including those that utilize attenuated virus, virus-like particles (VLPs), protein subunits, and nanoparticles, as well as recent RNA vaccines, with several of these approaches showing promise. Historically, vaccine candidates have advanced, dependent upon the ability to activate the humoral immune response, specifically leading to strong B cell responses and neutralizing antibody production. More recently, it has been recognized that the cellular immune response is also critical in proper resolution of viral infection and protection against detrimental immunopathology associated with severe disease and therefore, must also be considered when analyzing the efficacy and safety of vaccine candidates. These candidates would ideally result in robust CD4+ and CD8+ T cell responses as well as high-affinity neutralizing antibody. This review will aim to summarize established and new approaches that are being examined to harness the cellular immune response during respiratory viral vaccination.

## 1. Introduction

Respiratory infections are responsible for significant healthcare burden throughout the world largely due to the development of lower respiratory tract infections (LRTIs). LRTIs represent a leading cause of human mortality and morbidity, causing over 3 million deaths annually worldwide, making them the fifth leading cause of death overall and the leading cause of infectious death in children under the age of five [1]. Approximately 80% of LRTI cases are caused by viruses. Among the most prevalent are infections with respiratory syncytial virus (RSV), rhinovirus (RV), influenza virus, and more recently, severe acute respiratory syndrome coronavirus-2 (SARS-CoV-2). In the case of RSV, RV, and influenza, these infections are particularly problematic for infants. Children under one year of age account for 6.4 million instances of severe LRTI, with infants exhibiting a three-fold increase in the rate of fatality following infection compared to children > 12 months of age [2]. For RSV infection, approximately half of children requiring hospitalization are ≤3 months of age [3]. However, the elderly, immunocompromised individuals, and those with chronic conditions, including asthma, are also at high risk of developing LRTIs, making respiratory viral infections a significant cause of morbidity and mortality in these individuals [4,5]. Interestingly, infants, children, and young adults appear to be largely spared from the severe disease caused by SARS-CoV-2, whereas the elderly and high-risk (i.e., obese, diabetic, hypertensive) individuals are most susceptible to the detrimental effects of this infection. According to Global Burden of Diseases, 74% of deaths associated with LRTIs represent these vulnerable patient groups [1]. It is also important to note that throughout life, males are more susceptible to severe disease caused by respiratory viruses compared to females. In the case of early-life RSV, males are hospitalized at an approximately a 2:1 ratio compared to females due to lower respiratory tract diseases, such as bronchiolitis and pneumonia [6]. SARS-CoV-2 mortality is also much higher in males than in females, with men accounting for about 70% of deaths [7,8,9]. Therefore, there are likely sex-associated differences in not only response to infections but also vaccine responses that may need to be considered.

Long-term consequences of these respiratory viral infections have also been identified. For early-life infections, such as those caused by RV and RSV, an enhanced likelihood of developing childhood wheezing, including allergy and asthma, has been linked with severe disease [10,11,12,13]. According to the Centers for Disease Control and World Health Organization, severe COVID-19, the disease caused by SARS-CoV-2, has also been linked with many long-term side effects, including prolonged muscle and joint fatigue and general malaise as well as neurologic deficiencies and detrimental impacts on the cardiovascular system [14,15]. Therefore, prophylactic treatment to prevent severe respiratory illness caused by these viruses is imperative not only for reduction of infection and severity but also for protection against long-term disease pathologies throughout life. Targeting respiratory viral infections using current vaccine strategies has so far been unremarkable or, as with RSV, detrimental [16,17,18]. Additionally, while vaccines are well-established for influenza, widespread vaccination has yet to be achieved due to variability of response and sometimes sub-optimal protection as well as mistrust within the public regarding vaccination programs. It is crucial that vaccine strategies become more optimized and efficacious so that these obstacles may be overcome.

In this review, we will discuss the immune responses to respiratory viruses and how the cellular immune response may be harnessed in order to produce more promising vaccine candidates for viruses that have consistently been difficult to target. Utilizing these novel strategies will be crucial for developing these prophylactic treatments to protect against initial viral disease as well as protection from long-term consequences.

## 2. History of Vaccine Enhanced Disease

Under certain circumstances, a viral infection or vaccination may result in a subverted immune response, which may lead to an exacerbated illness. Clinical evidence of enhanced illness by pre-existing antibodies from vaccination, infection, or maternal passive immunity have been documented for many viruses [19]. Multiple mechanisms have been proposed to explain this phenomenon. It has been confirmed that certain infections and/or vaccine-induced immunity could exacerbate viral infectivity in Fc receptor or complement bearing cell-mediated mechanisms, leading to antibody-dependent enhancement (ADE) [19]. Another possible circumstance involves a condition referred to as enhanced respiratory disease (ERD), which may be caused by multiple mechanisms, including ADE [20]. These historical side effects have significantly hampered respiratory viral vaccine development and need to be addressed for any vaccine candidate and may be reduced by proper immune system response induction, including targeting both the cellular and humoral immune responses.

One of the most well-characterized and well-studied incidences of ERD is that which is associated with the failure of the formalin-inactivated RSV vaccine in the 1960s. Infants and toddlers immunized with this vaccine experienced an enhanced form of RSV disease characterized by high fever, bronchopneumonia, and wheezing when they became naturally infected with the virus [18,20]. Hospitalizations were frequent, and two immunized toddlers died upon infection with wild-type RSV. Decades of research have defined this as the result of immunization lacking proper toll-like receptor (TLR) signaling, with antigens not processed in the cytoplasm, resulting in a non-protective response that exacerbated immunopathology [20,21]. This response led to a pathogenic Th2 memory response, which is known to enhance secondary responses later in life [19,22,23,24]. At least part of this altered and disease enhanced response was likely due to the adjuvant itself, alum, known to drive inflammasome-induced activation via NLRP3-mediated response [25]. Thus, vaccine strategies for future success not only need to consider the virus components but also the route of vaccination and the adjuvant effect required for driving the proper immune responses.

## 3. Overview of Immune Response to Respiratory Viruses

Understanding immune responses to viral infections is crucial to progress in the quest for effective infection prevention and control. Host immunity involves various mechanisms to combat viral infections and requires the innate and adaptive immune system to lead to both cellular and humoral immune responses. Many respiratory viruses, including RSV, RV, influenza, and SARS-CoV-2, lead to strong cellular immune responses that are essential for control of not only the anti-viral response but also to inhibit prolongation of the inflammatory immune response, which in many cases is the etiological cause of severe disease.

### 3.1. T Cell Immunity

T cell-mediated immunity is a crucial part of the anti-viral immune response, largely through the induction of Th1-immunity and the production of the cytokine, interferon (IFN)-γ, as well as the CD8+ T cell cytotoxic response. T cells are activated by specific antigens presented by peptide-MHC complexes on the surface of antigen-presenting cells (APCs), such as dendritic cells (DCs). Next, these activated T cells are induced to clonally expand by the cytokine interleukin-2 (IL-2), and subsequently differentiate into effector T cells as a result of a specific subset of cytokines engaging and activating their respective cytokine receptors [26,27]. Distinct cytokine profiles reflect different T cell functions and include the effector CD4+ Th1-type, which primarily produce IFN-γ; helper CD4+ Th2-type characterized by the production of the cytokines IL-4, IL-5, and IL-13, and CD4+ Th17-type, which produce mainly IL-17 and IL-22. The virus-specific CD8+ T cells are directed by the type-I immune responses and differentiate to recognize and kill virally infected cells, influenced by both innate and acquired immune responses. The latter cytotoxic response is more crucial for respiratory viruses that release virions by budding from the surface of infected cells, as opposed to influenza, a lytic virus releasing virions in one explosive event. The overall role of T cells differs in that Th1 cells are mainly involved in cell-mediated immunity whereas Th2 cells are responsible for humoral immunity and B cell development of the antibody response [28,29,30]. The Th17 arm of the immune response is largely involved in antibacterial and mucosal immunity [31]. A successful immune response requires a balance of these subsets and inappropriate immune response skewing has been linked to the development of pathologic responses, such as that observed following RSV infection [32,33,34] that has exaggerated Th2 and Th17 with reduced Th1 responses. The imbalance of the Th cell responses away from the preferred Type-1 response leads to prolonged and exacerbated disease phenotypes, persisting long after the virion production has subsided. Furthermore, it is well known that CD4+ T cell help is important for optimal antibody responses and for CD8+ T cell activation in host defense [28,35,36]. In responses where neutralizing antibody-mediated protection is incomplete, cytotoxic CD8+ T cells are crucial for viral clearance [35,37]. Less severe cases of respiratory infections are associated with induction of a Th1 cell response [38,39], whereas Th2 cell responses have been associated with enhancement of lung disease following infection in hosts previously vaccinated against viruses, such as RSV and SARS-CoV [20,40,41].

### 3.2. Dendritic Cell Role during Respiratory Viral Infection

Dendritic cells (DCs) play a crucial role in the development of the immune response and link innate and adaptive immunity by instructing T cells toward a Th1 (anti-viral), Th2 (anti-parasitic or allergy-associated), or Th17 (anti-bacterial or autoimmunity) type response. DCs are sentinel cells present in the lung during steady-state conditions that constantly monitor the lungs for foreign pathogens or antigens. Upon infection, myeloid cells, including monocyte-derived DC (moDC) and inflammatory DC from the bone marrow, robustly expand and are recruited to the inflamed sites to clear pathogens or infected cells to support both the innate and adaptive immune responses. Following respiratory viral infection, DCs acquire viral antigen either through direct infection or indirectly from dying infected cells. They then undergo maturation and migrate to the lung-draining lymph nodes (LDLNs) where they present antigen to naive T cells. While there are numerous subsets of DCs, they can be largely divided into defined populations: moDCs, conventional DCs (CD103/CD11c; CD11c/CD11b), and plasmacytoid DCs (pDC). Plasmacytoid DCs are derived from the lymphoid lineage and promote a protective role during viral infection largely through the production of type-1 IFN [42,43]. Conventional CD11b+ and CD103+ DC within the lung are myeloid derived and have been implicated in pathogenic inflammatory responses during respiratory viral infection [42,44]. These latter DCs provide the direct link to acquired immunity through their APC function and specific innate cytokine responses that are directed by the virus infection itself. For the most appropriate viral response, DCs instruct T cells toward anti-viral Th1 via the production of instructive innate cytokines, such as IFN-β and IL-12, which leads to the production of IFN-γ and proper viral clearance [45]. However, many respiratory viral infections, such as RSV, RV, and SARS-CoV-2, have the ability to dampen type-1 immunity through multiple mechanisms [46,47,48,49,50,51] that lead to a decrease in T cell production of important anti-viral cytokines, such as IL-2 and IFN-γ. These skewed responses lead to an imbalanced immune response away from Th1 anti-viral immunity toward a predominantly Th2/Th17 pathogenic response accompanied by a diminished CD8+ cytotoxic T cell response. Thus, a critical balance is required between pDC and cDC for proper inflammatory and viral responses within the lungs and must be considered during vaccine development.

### 3.3. Trained Immunity

Trained immunity involves innate cells, including DC, and is characterized by non-specific responsiveness upon secondary stimuli exposure mediated through altered signals involving transcriptional, epigenetic, and metabolic pathways [52,53,54]. Trained innate immunity is distinguished from classical adaptive immunity by the ability to mount a different and sometimes much stronger transcriptional response when challenged with unrelated pathogens or other stimuli. These responses are likely influenced throughout life by nutritional, environmental, and microbial/viral interactions.

The phenomenon of trained immunity has been widely studied in recent years in relation to the tuberculosis vaccine, Bacille Calmette–Guérin (BCG) [52,55]. Previous vaccination with BCG has been linked to protection from unrelated pulmonary pathologies that reduce morbidity and mortality in vaccinated individuals [56,57]. Interestingly, during the recent SARS-CoV-2 pandemic, these findings are consistent for protection from severe COVID-19 in BCG vaccinated individuals upon SARS-CoV-2 infection [58,59,60]. Others have suggested that other type-1 promoting vaccines, such as measles, mumps, rubella (MMR), that contain live attenuated viral strains also may set up innate immune training and provide protection from future viruses, such as SARS-CoV-2 [61,62,63]. Furthermore, it has been suggested that boosted MMR vaccination of older individuals may offer protection from SARS-CoV-2, not only to enhance the trained immune response but also as direct protection since the rubella virus component of MMR has 29% sequence homology with SARS-CoV-2 [62]. *Haemophilus influenzae* and pneumococcal vaccines have also been suggested as offering protection from COVID-19 severity due to trained immunity responses [62,63,64]. Perhaps these trained immune responses to early-life vaccines may contribute to the protection observed in children during SARS-CoV-2 infections that wanes as we age. On the other hand, studies have linked inappropriate DC-specific trained immunity that leads to a decrease in type-1 IFN levels as a possible route for enhanced COVID-19 disease in susceptible populations [49]. These observations, along with other studies that have shown that DCs from naturally infected individuals have decreased sensitivity to future unrelated TLR signaling [65,66], suggest that proper activation of DCs and appropriate TLR targeting (i.e., to enhance Th1 immunity) during vaccination may lead to “training” of innate responses to future encounters with unrelated pathogens.

## 4. Novel Vaccination Approaches against Respiratory Viral Infections

Recent discoveries in immune response requirements for strong vaccine candidates have led to the development of many novel vaccine approaches that are currently being explored and showing remarkable success both in animal modeling studies as well as clinical trials (Table 1). These include, but are not limited to, nanoparticle-based vaccines, virus-like particle (VLP) vaccines, adenoviral vector constructs, and maternal immunization for early-life protection, which are further described below. While much of the discussion surrounds vaccination strategies for influenza, RSV, and SARS-CoV-2, many of these techniques may be employed for vaccine development against other viral pathogens, including RV and HMPV, that utilize similar immune evasion pathways.

### 4.1. Targeting T Cells during Vaccine Development

For many years, the focus of vaccines was to elicit neutralizing antibodies, but it has become increasingly evident that T cell-mediated immunity plays a central role in controlling respiratory viral infections, such as RSV, RV, HMPV, influenza, and coronaviruses. Targeting the cells in the local lung environment, including immune (DCs, T cells, B cells) as well as non-immune cells, may be crucial. For example, long-lived lung-resident killer T cells that recognize specific viruses and protect against re-infection. A recent study determined that tissue-resident memory T (Trm) cells could be reactivated by both immune as well as non-immune cells, such as DCs and epithelial cells [67]. Because these lung-resident cytotoxic Trm cells can be quickly reactivated by infected cells at the site of pathogen entry, identifying vaccines that can generate Trm cells will be critical for long-term immunity to viral infections of the lungs. While there are effective antibody-inducing viral and bacterial disease vaccines, more complex pathogens may require a dual approach that also involves the engagement of cytotoxic T cells. The development of T cell-inducing vaccines is rapidly expanding to induce CD4+/CD8+ T cells that directly contribute to pathogen clearance by cell-specific mechanisms. These T cells will need to be of the appropriate phenotype to generate protective (i.e., Th1) rather than immunopathogenic (i.e., Th2-skewed) responses.

In the earliest success of vaccination, including smallpox and rabies, a T cell response to the vaccine was induced and persists for many years but also added to the safety of the vaccine by protecting against immunopathology through proper Th1 immune skewing while still promoting neutralizing antibody for protection [68,69,70]. However, for the vaccine against tuberculosis (TB), BCG, a T cell response is essential for protection. Both CD4+ and CD8+ T cells are involved in protection against TB disease [68]. BCG may therefore be considered the first T cell-inducing vaccine, and still the only licensed vaccine promoting primarily T cell responses. In recent years, T cell-inducing vaccines have been evaluated for HIV, influenza, and malaria [37,68,71,72,73,74,75]. For example, a modified vaccinia virus Ankara (MVA) vector encoding the influenza nucleoprotein and matrix protein 1 (MVA-NP+M1) was tested in human clinical trials and showed a strong Th1 (IFN-γ) response with an acceptable safety profile [76]. An animal study that identified multiple influenza epitope targets used to vaccinate transgenic mice induced a CD8+ T cell response that was specific for HLA-A*0201, which also reacted in human cells infected with multiple unrelated influenza strains, confirming that these regions contain epitopes naturally occurring in humans that are conserved amongst non-related viruses [77]. Similar to these findings, a safety and efficacy clinical trial testing a six fluorocarbon-modified 35-mer peptide encapsulating multiple CD4+/CD8+ T cell epitopes against pan-influenza showed significant T cell-specific responses and acceptable safety profiles in the majority of test subjects [78]. In another clinical trial, Multimeric-001, containing conserved hemagglutinin (HA), NP, and M1 epitopes, designed to protect against seasonal and pandemic influenza indicated significant IFN-γ production when sera from vaccinated individuals was exposed to H1N1, H3N2, or influenza B viral strains in the Madin–Darby canine kidney (MDCK) cell lysis model [79]. Importantly, these studies suggest that employing vaccination strategies to incorporate multiple T cell epitopes may not only be successful in driving strong T cell-mediated immunity but may also mitigate the need for yearly influenza vaccinations due to their ability to protect against numerous viral strains. Given the strong role of the T cell response in respiratory viral infections, vaccinations that target these cells will be important, while still eliciting appropriate neutralizing antibody responses.

The current successful human anti-viral vaccines, such as influenza and measles, depend largely on the induction of antibody responses; however, emerging evidence suggests the requirement of both antibody- and T cell-mediated immunity for effective protection against respiratory viruses, including RSV, RV, HMPV, and SARS-CoV-2, as well as influenza as described above. Interestingly, a study by Mateus et al. showed that SARS-CoV-2-reactive CD4+ T cells are reported in up to 50% of individuals that have never been exposed to the virus, suggesting pre-existing T cell memory [80]. These T cells include a variety of cross-reactive memory CD4+ T cells that react to SARS-CoV-2 as well as common cold coronaviruses with similar levels of affinity. This suggests that T cell memory to common cold coronaviruses may limit COVID-19 disease severity and further promotes the inclusion of multiple T cell epitopes within vaccine design. Furthermore, people who have recovered from COVID-19 have high levels of both neutralizing antibodies and T cells, with milder cases of COVID-19 having greater numbers of virus-specific memory CD8+ T cells in the respiratory tract, further supporting a significant role of Trm cells for protection against disease [68,81,82]. These preferred T cell responses may be further achieved by proper adjuvanted vaccines to drive appropriate responses. For example, infant mice given an alum-adjuvanted RSV F antigen vaccine led to a Th2 skewed response, whereas animals given an Advax-SM adjuvant had both neutralizing antibody and protective non-Th2 type immune responses and reduced immunopathology [83].

### 4.2. Importance of Proper TLR/APC Signaling and Vaccine Adjuvanticity

Targeting the APCs that are crucial for guiding the T cell response will also be necessary for a strong vaccine candidate. TLR engagement in APCs, such as with DCs, leads to the production of cytokines that instruct the T cells towards Th1 inducing (type 1 IFN, IL-12). Inefficient activation of TLR or other PRRs can lead to an inappropriate response characterized by Th2-inducing (low type 1 IFN, IL-12) or Th17-inducing (IL-6, IL-23) phenotypes. For strong Th1 induction, engagement of TLR3, TLR4, or TLR7 leads to high levels of IFN-α/β and IL-12, which drive T cells to express IFN-γ. These innate cytokines not only promote anti-viral activity but also inhibit the Th2 response; therefore, engaging this pathway during vaccination by proper TLR adjuvancy may significantly increase the efficacy and safety of these vaccine candidates. Furthermore, studies have shown that by simply targeting DCs, RSV-induced Th2-driven immunopathology is reduced [84,85], indicating that the DC itself can be modified to limit inappropriate T cell skewing during respiratory viral infection, which may extend to vaccine protection.

Recently, vaccination efficacy and safety studies have been evaluated using TLR targeting with promising results. The TLRs that have been widely used in vaccine adjuvants are: TLR3, 4, 5, 7, 8, and 9 [86,87]. Out of these, only the TLR4 ligand monophosphoryl lipid (MPL) has been approved for use in human vaccine formulations, such as Human papillomavirus vaccine (Cervarix), hepatitis (Fendrix), and malaria (RTS, S/AS01, or Mosquirix) [87]. However, TLR adjuvants have shown promising results in respiratory virus vaccine studies and require further discussion. For example, one study found that inclusion of the TLR4 ligand monophosphoryl lipid A (MPLA) in reconstituted RSV membranes (virosomes) potentiated vaccine-induced immunity and skewed the immune responses toward a Th1 phenotype, without priming for ERD [88] as with alum adjuvanted RSV vaccines, suggesting a safer more effective vaccination strategy. Incorporation of Pam3CSK4, a TLR2 agonist, increased the capacity of virosomes to activate APCs in vitro and boosted serum IgG antibody responses and mucosal antibody responses after immunization and protected mice against infection with RSV, without priming for enhanced disease [89]. Studies have also shown that TLR9 activation can protect against vaccine-enhanced disease [90]. Vaccine formulations with the inclusion of TLR7/8 agonists, such as imiquimod, have historically been difficult to deliver due to their small molecular size; however, recently, the incorporation of these molecules into new delivery systems, such as nanoparticles, has garnered some success [91]. Together, these studies support the idea that proper TLR-activating adjuvants and APC targeting may significantly change the landscape for respiratory viral vaccination towards safer and more efficacious options.

Importantly, extensive efforts around the globe are being made to develop a suitable vaccine against SARS-CoV-2. Incorporating a suitable adjuvant in a SARS-CoV-2 vaccine may help facilitate this discovery. TLR adjuvants have been widely studied using other coronaviruses [86,87,92,93,94,95] and may help guide vaccine development for the novel SARS-CoV-2. Using vaccine studies with the previous SARS-CoV strain as a guide, researchers have been able to accelerate the pace of development with the more infectious SARS-CoV-2 strain. As with that seen in the formalin-inactivated RSV individuals, inactivated SARS-CoV vaccine with alum adjuvant has been reported to cause eosinophil infiltration in the lungs of immunized animals following live viral challenge, but TLR3 and 4 ligand (poly(I:C) and lipopolysaccharide) inclusion into the vaccine formulation was able to overcome this issue [92]. In another study, intranasal administration of a poly(I:C)-adjuvanted vaccine induced IFN-β and IFN-γ production and protected animals from respiratory viral infections caused by influenza and SARS-CoV [93]. Studies have identified the TLR7 pathway as more activated in females compared to males following SARS-CoV-2 infection and linked to protection from severe disease [49], suggesting that viral stimulation through TLR7 may lead to a stronger type-1 IFN response in females and that adjuvant targeting of this pathway may be beneficial, especially in males. Mice immunized with inactivated SARS-CoV and the TLR9 ligand, CpG, also showed promising results [94]. Another study showed that CpG induced a memory T cell phenotype that conferred long-term responsiveness [95]. Further, much like other respiratory viruses, there are concerns that the SARS-CoV-2 spike protein subunit vaccine adjuvanted only with alum, which favors strong Th2 responses, might trigger immunopathology in the lungs; therefore, the inclusion of a TLR ligand that favors Th1 may lead to a reduction in ADE as well as activation of both arms of the immune system. While a strong Th1-driving adjuvant is preferred against most respiratory viral pathogens, it will undoubtedly be necessary to induce a balanced immune response along with neutralizing antibody and possibly the induction of other T cell-mediated pathways, such as Th17, to avoid over-activating one arm of the immune system, which may itself lead to unwelcome side effects. With numerous advances in the vaccine field, a wide range of novel approaches have been taken in the past year.

### 4.3. Current Vaccination Strategies

#### 4.3.1. Nanoparticle-Based Vaccines

Conventional vaccines have historically come with risks, such as pathogenic responses to live-attenuated vaccines or weak immune responses observed with inactivated pathogen vaccines, and subunit vaccines were developed to compensate for these issues. However, subunit vaccines may confer limited immunogenicity and often only induce partial protection as is observed with the pertussis subunit vaccine [96]. To address limitations caused by both conventional and subunit vaccine platforms, nanoparticle-based vaccines have been developed and show great potential [97]. Currently, many types of nanoparticles are being evaluated, including virus-like particles (VLPs) and liposomes, as well as inorganic, polymeric, and self-assembled protein nanoparticles [97]. Since many biological systems, including viruses and proteins, are “nano-sized”, the similar size of these nanoparticles leads to enhanced antigen presentation and subsequently stronger immunogenicity. Studies have shown that nanoparticles protect antigen structures from proteolytic degradation and/or have the ability to improve antigen delivery to APCs [98]. Animal modeling studies have shown promising results using nano-sized adjuvants during vaccine development. For example, a nanoscale oil-in-water emulsion adjuvant that produces strong Th1- and Th17-protective immune responses to a variety of antigens [99,100,101] is a promising candidate for this type of delivery system. In fact, the use of this adjuvant in RSV vaccination induced Th1/Th17 immunity, protected against viral challenge in two different animal models (mouse and cotton rat), and ameliorated virus-induced Th2-associated pathology, which is known to lead to ERD [99,101].

SARS-CoV-2 vaccine candidates are progressing at an unprecedented speed and many of these candidates include nanoparticle designs, including the Pfizer (BNT162) and Moderna (mRNA-1273) RNA vaccines that are currently in clinical trials and showing promising results [102,103,104,105,106]. BNT162, a lipid nanoparticle-formulated nucleoside-modified mRNA that encodes the receptor binding domain (RBD) of the SARS-CoV-2 spike protein, is now in phase III clinical trials and purported to show >90% efficacy in protection against infection [107]. Early phase I/II safety and efficacy studies showed that BNT162 indicated transient mild to moderate local reactions and systemic events that were dose dependent and led to RBD-binding IgG concentrations and SARS-CoV-2 neutralizing titers in sera that increased with the dose level and after a second dose [102]. A separate phase I/II study indicated that immune sera from vaccinated patients broadly neutralized pseudoviruses with diverse SARS-CoV-2 spike variants. Importantly, most participants had Th1-skewed T cell responses, with RBD-specific CD8+ and CD4+ T cells releasing high levels of IFN-γ [103]. The mRNA-1273 vaccine being manufactured by Moderna, also in phase III testing, shows similar safety and efficacy profiles as BNT162 but has shown an added benefit of protecting older adults (>56 years old) with high Th1-skewed T cell responses [104,108]. As we finalize this review, both Pfizer and Moderna RNA vaccines are rapidly moving toward approval, with reported efficacy of ~95% in phase III clinical trials. Thus, the early results suggest that these vaccine candidates provide a desired immune phenotype, potentially indicating that the lipid containing RNA promotes a proper innate immune environment through multiple beneficial innate and acquired immune mechanisms.

#### 4.3.2. Virus-Like Particle Vaccines

Virus-like particles (VLPs) are multiprotein structures that mimic the organization and conformation of authentic native viruses but lack the viral genome, potentially yielding safer and cheaper vaccine candidates. A number of VLP-based vaccines are currently marketed, including Engerix (hepatitis B virus), Cervarix (human papillomavirus), Recombivax HB (hepatitis B virus), and Gardasil (human papillomavirus) [109]. Other VLP-based vaccine candidates are in clinical trials or undergoing preclinical evaluation, such as influenza virus, parvovirus, Norwalk, and various chimeric VLPs [109]. Additionally, studies have been focusing on VLPs for RSV vaccine candidates [110,111,112]. Mice immunized with RSV VLPs (RSV-F or RSV-G protein) showed higher viral neutralizing antibodies in vitro and significantly decreased lung viral loads in vivo after live RSV challenge [111]. In another study, RSV-F VLP induced protection in mice without causing pulmonary RSV disease by inducing RSV neutralizing antibodies, as well as modulating specific subsets of DC and CD8+ T cell immunity [112]. Upon a challenge infection, a significantly lower viral load was measured in the lungs of mice immunized with RSV-G VLPs compared to naïve and formalin-inactivated RSV-immunized control mice, along with increased memory B cell responses in the spleen and significantly decreased inflammation in the lungs [110]. These studies suggest that VLP-based vaccines could provide promising vaccine candidates against RSV and other respiratory viruses.

VLP vaccine candidates are also being evaluated for coronavirus vaccination, including SARS-CoV-2 [113,114,115]. One study showed that VLPs transfected with three mRNAs encoding SARS-CoV-2 S (spike), M (membrane), and E (envelope) encapsulated in lipid nanoparticles elicited both humoral and T cell immune responses in mice with higher neutralizing antibody titers compared to an S mRNA nanoparticle vaccine, which only showed humoral immunity, suggesting superiority in the VLP vaccine candidate [113]. In a vaccine candidate for Middle East respiratory syndrome (MERS), poly(I:C), a TLR3 adjuvant, was superior to alum when administered to mice with chimeric VLPs expressing MERS-CoV RBD protein by eliciting stronger neutralizing antibody as well as cell-mediated responses that prevented virus entry into susceptible cells [116]. Given these data and the lack of type-1 IFN production during SARS-CoV-2 infection and correlation with severe disease, including such adjuvants that are likely to enhance type-1 IFN production, such as TLR3/4 or TLR7, may be crucial, especially for vaccination of the elderly and immunocompromised patients.

#### 4.3.3. Adenoviral Vector Vaccines

Adenoviral vector (AdV) vaccines have been in development and deployed for human use against infectious disease as well as cancer since the 1970s and have been widely suggested for use against severe outbreaks, such as Ebola, Lassa fever, Nipah, and MERS [117,118]. More recently, AdVs have been widely applied as vaccine carriers because they elicit both T and B cell responses, leading to cellular and humoral immunity [118]. They are generally considered safe, are easy to replicate, and can be administered through multiple routes, including oral, intranasal, or intramuscular, and do not require adjuvants. Many different species may be utilized to isolate AdVs, including human, bovine, and chimpanzee. Despite being studied for decades, AdV-incorporated vaccines have not been widely approved for use partially because adenoviruses are a common source of natural exposure to humans and pre-existing neutralizing antibodies may lead to decreased immunogenicity as the immune response eliminates the AdV before immunity can develop against the desired pathogen. Chimpanzee-derived AdVs (ChAd) is being used to circumvent this issue and has been widely studied in the recent SARS-CoV-2 ChAd (ChAdOx1 nCoV-19) vaccine program being developed at Oxford and was, in fact, one of the first SARS-CoV-2 vaccine candidates to enter clinical trials [119,120]. There are now five SARS-CoV-2 vaccine candidates in clinical trials that employ adenovector-based vaccine strategies, including three using human adenovirus [121,122,123] and two utilizing non-human primate (chimpanzee and gorilla) [120]. Results from two human adenovirus candidates and ChAdOx1 nCoV-19 are showing promising results with low adverse events and strong antibody responses as well as Th1/INF-γ-producing CD4+/CD8+ T cells without Th2 skewing [120,121,122]

Evaluation of AdV vaccines for RSV and influenza has been ongoing for many years and has garnered success but have not yet been approved for human use. An RSV vaccine candidate utilizing the human adenovirus serotypes 26 and 35 encoding the F fusion protein of RSV was tested in mice and cotton rats and indicated high neutralizing antibody levels and F-specific IFN-γ-producing Th1 cells that protected against live viral challenge as well as vaccine-enhanced disease [124]. An additional study showed that a gorilla-based AdV-RSV F fusion-based vaccine can generate strong antibody and T cell responses to protect against live RSV challenge as well as diminished Th2-type responses [125]. Immunization of multiple species, including non-human primates, with PanAd3-RSV, a chimpanzee-derived AdV encoding RSV F, N, and M2-1 proteins, led to strong neutralizing antibodies and broad T cell immunity [126]. Furthermore, this PanAd3-RSV vaccine candidate was tested using infant bovine calves and induced both humoral and cellular immunity to protect against live RSV and enhanced respiratory disease [127]. These studies led to the successful transition to a phase I clinical trial that indicated that PanAd3-RSV, as well as a MAV-RSV candidate, can boost neutralizing antibody titers as well as generate strong CD4+/CD8+ IFN-γ-producing T cells without priming for Th2 cell expansion [128]. A ChAd-155-RSV F was also recently tested in a phase I clinical trial and showed similar results [129]. AdV-based influenza vaccines have also been widely studied and a comprehensive review was recently published [130]. In short, studies have evaluated the protective effect of AdV-based influenza vaccines in animal modeling and completed clinical trials. Most of these studies utilized the human AdV serotype 5 (hAdV5), which promotes both cellular and humoral immune responses, leading to protection in mice, poultry, pigs, and ferrets when used to deliver influenza epitopes (HA, HA1, HA2, NP, M2) [130,131,132]. Multiple other adenovirus platforms, including ChAd- and bovine adenovirus-based vaccines, have also been tested in preclinical influenza studies, showing effective results [130]. Importantly, influenza hAdV-based vaccines have also been tested in clinical trials up to phase IIb and have so far shown acceptable safety profiles and strong humoral immunity [130]. These studies suggest viral vector vaccines as a promising platform for respiratory virus vaccine development.

#### 4.3.4. Maternal Vaccination for Early-Life Prevention

Respiratory viral infections are a leading cause of mortality and complications in infants. As mentioned above, children under one year of age account for 6.4 million instances of severe disease and exhibit a three-fold increase in the rate of fatality following infection compared to children > 12 months of age [2]. Thus, vaccination of infants is an important strategy for minimizing disease. However, it is proven very difficult to vaccinate infants for multiple reasons, including an underdeveloped immune system and a predisposed environment for Th2-type responses, which are known to cause severe respiratory disease [133,134,135]. Additionally, the timing of vaccination in infants when multiple doses are required is very difficult. For example, with RSV infection, approximately half of children requiring hospitalization are ≤3 months of age [3], indicating that proper vaccination and the development of protective immunity needs to occur shortly after birth. In recent years, the concept of maternal vaccination has arisen to overcome these obstacles, with the idea that protection will be transferred from the mother to the infant in utero.

Animal models using this maternal immunization strategy have shown some promise. Maternal immunization for protection against influenza was observed in ferret models; however, the level of protection depends upon the number of doses used to vaccinate the mother, the presence of adjuvant, whether or not the mothers were primed by prior infection, and the age of the neonate at challenge [136]. Neonatal ferrets were passively immunized following maternal vaccination with formalin-inactivated influenza A virus vaccine and were completely protected up to 2 weeks of age, but susceptibility returned at 5–7 weeks of age. A recent cotton rat study assessed the efficacy of VLP vaccine candidates containing stabilized pre-fusion (pre-F) or post-fusion (post-F) conformations of the RSV F protein and the attachment RSV G protein in a maternal immunization model [137]. This study determined that VLP vaccines containing RSV F and G proteins strongly boosted pre-existing RSV immunity in dams as well as providing significant protection to pups from RSV challenge and reduced pulmonary inflammation. On the other hand, maternal vaccination of cotton rats using a formalin-inactivated RSV vaccine showed enhanced disease in pups given an RSV infection at 1 or 4 weeks of age [138]. Additionally, pups that were born to non-vaccinated dams that were either RSV infected or uninfected, and given this same formalin-inactivated vaccine at 4 weeks of age followed by an RSV infection 4 weeks later, showed enhanced disease, regardless of whether the mother was seronegative or seropositive. These studies reinforce that success in vaccination against respiratory viral infections will require proper delivery methods and adjuvant modeling.

While the concept of maternal vaccination seems feasible and promising, the logistics and procedures for success in humans are of significant concern. Pregnant women must be vaccinated at the appropriate time to generate enough antibody in utero to lead to protection and this must be timed precisely to ensure that this is accomplished prior to birth, especially in infants being born during peak virus season. A recent clinical trial attempted maternal vaccination against neonatal RSV infection using a subunit RSV-F protein vaccination strategy and showed disappointing results [139]. Overall, the vaccination did show correlates of protection, including a decrease in the percentage of infants with RSV-associated medically significant LRTI from 2.4% in the placebo group to 1.5% in the vaccine group with the percentages for hospitalization decreased from 3.7% to 2.1%, but the predetermined criteria for efficacy was not achieved. For influenza vaccination, a recent population-based study determined that infants of vaccinated mothers were 45–48% less likely to have influenza hospitalizations than infants of unvaccinated mothers [140], indicating maternal vaccination against influenza as a positive model for infant protection. Together, these human studies indicate that some level of protection can be achieved through maternal vaccination and warrants further exploration for respiratory virus protection of infants.

## 5. Conclusions

**Figure 1:**
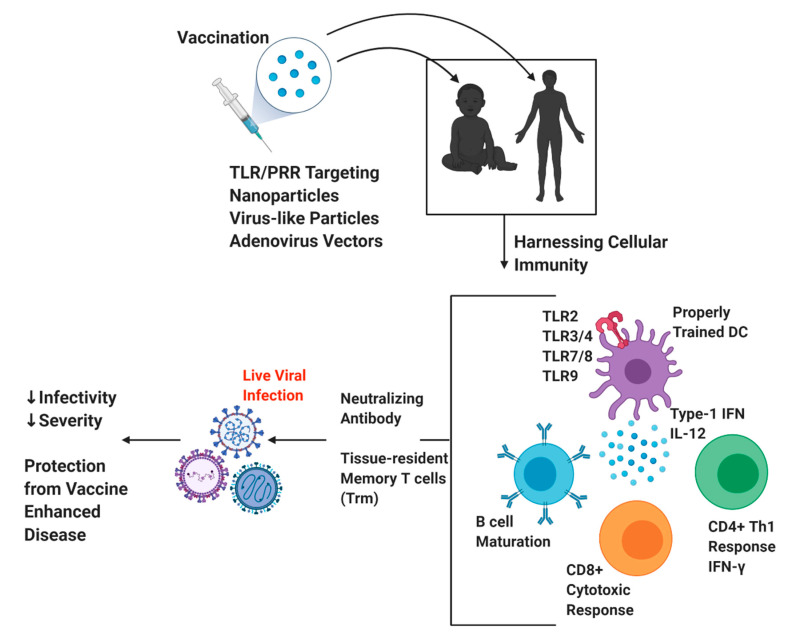
Overview of Harnessing Cellular Immunity for Respiratory Virus Vaccine Development. Figure created using https://biorender.com/.

Severe disease caused by respiratory viruses continues to be a significant healthcare burden and cause of morbidity and mortality worldwide [4,5,141,142]. In addition, emerging viral infections, such as the SARS-COV-2, will require new strategies to quickly and efficiently develop vaccines that are protective and safe. Vaccination against respiratory viruses have been sub-optimal due to lack of activation of appropriate innate trained immunity to drive proper humoral and cellular responses to avoid the threat of vaccine-enhanced disease. Inclusion of TLR or other PRR agonists as potent stimulators of DC maturation to instruct T and/or B cell activation is an attractive approach (Figure 1). This review has highlighted exciting aspects of targeting the cellular immune response to enhance vaccine efficacy and safety against respiratory viral infections. The development of a successful vaccine candidate will be informed by previous studies to lead to improved protection from initial infection as well as to decrease vaccine-enhanced diseases.

## Figures and Tables

**Table 1:** Overview of Vaccination Strategies.

General Vaccine Strategy	Antigen (Respiratory Virus Target)	Candidate Vaccine Examples	Induced Immune Response	Review Location
T cell epitope	MVA vector encoding NP, M1 subunit (Influenza)	MVA-NP+M1	CD8+ IFN-γ-producing T cells	Section 4.1
	Multiple Influenza T cell epitopes (Influenza)	Preclinical	HLA-A*0201 Cross-reactive CD8+ T cell responses	Section 4.1
	Multiple CD4+/CD8+ T cell epitopes (Influenza)	FP-01.1	Dual CD4+/CD8+ T cell responses and vaccine-specific T cells that cross react with multiple divergent influenza strains	Section 4.1
	HA, NP, M1 protein subunit (Influenza)	Multimeric-001	Th1/IFN-γ-driven protection against H1N1, H3N2, and influenza B	Section 4.1
TLR-adjuvant	Virosomes + TLR4 (RSV)	Preclinical	Th1 response without Th2 skewing	Section 4.2
	Virosomes + TLR2 (RSV)	Preclinical	Activated APC and Th1 response without Th2 skewing	Section 4.2
	Formalin-inactivated RSV + TLR9 (RSV)	Preclinical	Increased Th1 cytokine response with decreased Th2; protection from vaccine enhanced disease	Section 4.2
	UV-inactivated SARS-CoV + TLR3/4 (SARS-CoV)	Preclinical	Reduction of immunopathogenic Th2 responses	Section 4.2
	TLR3 pretreatment (Influenza and SARS-CoV)	Preclinical	Upregulation of IFN-β and IFN-γ production	Section 4.2
	SARS-CoV S peptide subunit + TLR9 (SARS-CoV)	Preclinical	Induction of IFN-γ-producing CD8+ memory T cells	Section 4.2
Conventional	Live-Attenuated (Multiple)	Multiple	Highly immunogenic but may lead to pathogenic immune responses	Section 4.3
	Inactivated Whole Virus (Multiple)	Multiple	Weak immune response without the addition of adjuvant	Section 4.3
	Subunit (Multiple)	Multiple	Limited immunogenicity without proper adjuvancy or packaging (i.e., nanoparticle, virus-like or live viral vectors)	Section 4.3
Nanoparticle	mRNA/ RBD spike (S) protein subunit (SARS-CoV-2)	BNT162	CD4+/CD8+ IFN-γ-producing T cells	Section 4.3.1
	mRNA/ pre-fusion S-2P (SARS-CoV-2)	mRNA-1273	Strong CD4+ Th1 cell response, low level CD8+ T cell response	Section 4.3.1
Virus-like Particles (VLP)	RSV-F subunit (RSV)	Preclinical	Induces CD8+ and CD103+ DC and F-specific IFN-γ/TNF-α CD8+ T cells	Section 4.3.2
	mRNA/ S, M (membrane), and E (envelope) subunit (SARS-CoV-2)	Preclinical	CD4+/CD8+ IFN-γ-producing T cells	Section 4.3.2
	Chimeric/ MERS RBD and CPV + TLR3 adjuvant (MERS-CoV)	Preclinical	DC activation and Th1/IFN-γ, Th2/IL-4 production by splenocytes	Section 4.3.2
Adenoviral Vector	S protein (SARS-CoV-2)	ChAdOx1 nCov-19	Th1/INF-γ-producing CD4+/CD8+ T cells with no Th2-skewing	Section 4.3.3
	Pre-fusion S protein (SARS-CoV-2)	Ad26.COV2.S	Th1/INF-γ-producing CD4+/CD8+ T cells with no Th2-skewing	Section 4.3.3
	RBD subunit (SARS-CoV-2)	Ad5-COVID-19	Th1/INF-γ-producing CD4+/CD8+ T cells with no Th2-skewing	Section 4.3.3
	RSV F fusion subunit (RSV)	Preclinical Human Ad26/35	F-specific IFN-γ producing T cells	Section 4.3.3
	RSV F fusion subunit (RSV)	Preclinical Gorilla AdV	RSV-specific CD4+/CD8+ Th1 cells with limited Th2 responses	Section 4.3.3
	RSV F, N, and M2-1 subunit (RSV)	PanAd3-RSV Chimpanzee AdV	CD4+/CD8+ IFN-γ-producing T cells without Th2 priming	Section 4.3.3
	RSV F fusion (RSV)	ChAd-155-RSV F	CD4+/CD8+ IFN-γ-producing T cells without Th2 priming	Section 4.3.3
	Numerous HA, NP, and M2 protein subunits (Influenza)	Preclinical Human AdV5, ChAd, and bovine AdV Clinical: Multiple Human AdV	Broad humoral and cellular immune responses	Section 4.3.3
Maternal Vaccination	Formalin-inactivated influenza A (Influenza)	Preclinical	Limited humoral response in neonatal offspring	Section 4.3.4
	RSV F and RSV G protein subunit VLPs (RSV)	Preclinical	Neonatal protection with reduced vaccine enhanced disease	Section 4.3.4
	Formalin-inactivated RSV (RSV)	Preclinical	Enhanced disease of offspring upon live viral challenge	Section 4.3.4
	RSV-F nanoparticle subunit (RSV)	Phase III clinical trial	Decreased LRTI and hospitalization in infants born to vaccinated mothers	Section 4.3.4
	Influenza vaccination	Population-based study	Infants born to vaccinated mothers 45–48% less likely to become hospitalized	Section 4.3.4

AdV = Adenoviral Vector; CPV = Canine Parvovirus; HA = Hemagglutinin; LRTI = Lower Respiratory Tract Infections; M1 = Matrix Protein 1; M2 = Matrix Protein 2; MVA = Modified Vaccinia virus Ankara; NP = Nucleoprotein; RBD = receptor binding domain; TLR = Toll-like Receptor.
